# Cost-effectiveness of a mindfulness-based mental health promotion program: economic evaluation of a nonrandomized controlled trial with propensity score matching

**DOI:** 10.1186/s12889-019-7585-4

**Published:** 2019-10-17

**Authors:** Gerhard Müller, Manuela Pfinder, Christian Schmahl, Martin Bohus, Lisa Lyssenko

**Affiliations:** 1 AOK Baden-Württemberg, Baden-Württemberg, Germany; 2 Department of General Practice and Health Services Research, Heidelberg University Hospital, Heidelberg, Germany; 3 Department of Psychosomatic Medicine and Psychotherapy, Medical Faculty Mannheim, Heidelberg University, Mannheim, Germany; 4 Institute for Psychiatric and Psychosomatic Psychotherapy, Central Institute of Mental Health, Mannheim, Heidelberg University, Mannheim, Germany; 5 Department of Health, Antwerp University, Antwerp, Belgium; 6 Department of Public Health and Health Education, University of Freiburg, Freiburg, Germany

**Keywords:** Cost-effectiveness, Prevention, Mental health, Health promotion, Implementation research

## Abstract

**Background:**

Mental health promotion programs have been shown to reduce the burden associated with mental distress and prevent the onset of mental disorders, but evidence of cost-effectiveness is scarce.

**Objective:**

To evaluate the cost-effectiveness of a mindfulness-based mental health prevention program provided by health coaches in a multi-site field setting in Germany.

**Methods:**

The single-study based economic evaluation was conducted as part of a nonrandomized controlled trial, comparing the effects of a group-based prevention program to usual care based on propensity score matching. Participants (*N* = 1166) were recruited via a large statutory health insurance fund. Health outcome was assessed with the Hospital Anxiety and Depression Scale (HADS). Cost outcomes were actually incurred costs compiled from the health insurance’ records. Incremental cost-effectiveness ratios (ICER) were analyzed from a societal and a health care perspective for a 12-month time horizon with sampling uncertainty being handled using nonparametric bootstrapping. A cost-effectiveness acceptability curve was graphed to determine the probability of cost-effectiveness at different willingness-to-pay ceiling ratios.

**Results:**

From a societal perspective, prevention was cost-effective compared to usual-care by providing larger effects of 1.97 units on the HADS (95% CI [1.14, 2.81], *p* < 0.001) at lower mean incremental total costs of €-57 (95% CI [− 634, 480], *p* = 0.84), yielding an ICER of €-29 (savings) per unit improvement. From a health care perspective, the incremental health benefits were achieved at additional direct costs of €181 for prevention participants (95% CI [40, 318], *p* = 0.01) with an ICER of €91 per unit improvement on the HADS. Willingness-to-pay for the prevention program to achieve a 95% probability of being cost-effective compared to usual-care, was estimated at €225 per unit improvement on the HADS score from a societal, and €191 from a health care perspective respectively. Sensitivity analyses suggested differential cost-effect-ratios depending on the initial distress of participants.

**Limitations:**

Due to the complexity of the field trial, it was not feasible to randomize participants and offer an active control condition. This limitation was met by applying a rigorous matching procedure.

**Conclusions:**

Our results indicate that universal mental health promotion programs in community settings might be a cost-effective strategy to enhance well-being. Differences between the societal and health care perspective underline the call for joint funding in the dissemination of preventive services.

**Trial registration:**

German Clinical Trials Registration ID: DRKS00006216 (2014/06/11, retrospective registration).

## Background

Mental disorders account for a large percentage of the total burden of illness and constitute a major economic challenge in industrialized countries [1]. Excluding the costs of neurological disorders, the estimated yearly costs in Europe amounted to €418 billion in 2010 [2, 3] with 35% direct health care costs, 12% direct non-medical costs, and 53% indirect costs. In the United States, in 2013, mental disorders topped the list of the most costly conditions, with national health spending at $201 billion [4]. Developing effective treatments is only one option for reducing these costs and the individual burden. In the case of major depression, for example, studies have shown that existing treatments only reduce the burden of disease by approximately 35% [5]. Consequently, preventing mental disorders and enhancing mental health has become a global priority [6].

A variety of studies have shown attractive cost-effectiveness ratios for selective and indicated interventions, i.e., programs directed at participants at risk or with emerging symptoms (e.g. [7]). Most indicated programs for adults target the prevention of depression, are based on cognitive-behavioral (CBT) approaches, and apply minimal contact therapy [8], bibliotherapy [9, 10], internet-based CBT [11–13] or personalized interventions [14] after screening for depression. All of these studies have yielded willingness-to-pay ratios of less than $30,000 to $50,000 per quality-adjusted life year (QALY) gained, which is currently accepted by policymakers as a cost-effective treatment for mental disorders [15]. Despite these encouraging results, available prevention strategies are rarely implemented in routine public health services (e.g. [16, 17]).

Universal health promotion programs directed at entire populations have some specific advantages that might facilitate larger scale implementations (e.g. [18]). Most programs are deliverable without the need for highly trained professionals, enhancing applicability in a variety of settings and regions. Such approaches are potentially considerably less costly because they do not require screening for the eligibility of participants. Furthermore, such programs are easily accessible to a larger public, thus reaching individuals who might not seek assistance for fear of stigmatization or negative consequences [17]. Effectiveness of universal prevention of mental disorders has been shown meta-analytically for interventions delivered at the workplace [19], higher education facilities [20] and schools (e.g. [21, 22]), but cost-effectiveness has only rarely been addressed [23–25]. To our knowledge, there is no published cost-effectiveness data of universal prevention programs directed at mental health for the general adult population.

In this article, we present the economic evaluation of a community-based health promotion program, which has been implemented in 2014 across Baden-Wuerttemberg, one federal state of Germany, by a statutory insurance fund [26]. Main objective of the study was to investigate what effects can be achieved with a group-based universal prevention program in a real-world community setting. The clinical evaluation implies a significant overall reduction of emotional distress after three months, as well as at the one-year-follow-up [27, 28]. In the 12-month follow-up, new cases of psychopathological symptoms were prevented in 1 of 16 participants [28]. Here we examine cost-effectiveness both from a societal and health care perspective for a 12-month time horizon.

## Methods

The economic evaluation of the Life Balance health promotion program was planned and conducted in combination with the evaluation of the clinical effectiveness in a multi-site field-setting in Germany (trial registration ID: DRKS00006216, WHO International Clinical Trials Registry; approved by the ethical review committee at the University of Heidelberg, 2013620NMA). The study was designed as nonrandomized comparison to usual care based on propensity score matching. Data collection covered the period from November 2013 to August 2015 with a 12-month time-horizon for the cost-effectiveness analyses. Full details of the study design are discussed elsewhere [26–28]. The economic evaluation is reported in agreement with the Consolidated Health Economic Evaluation Reporting Standards statement [29].

### Setting and location

Design and methods of this evaluation study are substantially shaped by structural aspects of the German health care system, namely the health insurance obligation. Every citizen with an annual income below € 60,000 is legally obliged to hold a membership in a certified statutory health insurance fund. The funds are multi-payer financed and based on the principle of solidarity, whereby membership fees and benefits are independent of the individual health condition – resulting in a relatively equitable accessibility to health care services. Aside the compensation of any kind of medical treatment costs, these funds are one of the major providers for preventive services in Germany.

The present study was conducted in close cooperation with one of the major statutory health insurance funds, the “AOK Baden-Württemberg”, which facilitated the reach of a large target population and the collection of actually incurred costs. For implementation, a total of 240 health coaches employed at the fund were trained to deliver the program at 80 different established health centers throughout the state. The program was promoted in the context of a region-wide mental health campaign via various channels, and offered free of charge to all adult members of the insurance fund, comprising about 40% of the statutorily insured population of this particular federal state – which corresponds to a target population of approximately 4.4 million persons.

### Intervention

Life Balance is a group-based universal mental health promotion program for the general public with the aim to enhance general protective factors for mental health. Participants meet in groups of 10 to 14 persons for six weekly sessions á 90 min and an additional booster session about one month after completion. In order to facilitate the widespread implementation and longer-term availability of the program, sessions are conducted by health coaches following a structured manual. Basic requirement for being trained in this program were holding a degree in sports, nutrition or other health-related discipline and experience in delivering preventive health interventions. All health coaches received 5 days of training and supervision.

The content of the program comprises the topics mindfulness, compassion, personal values, social support networks and behavior change and are conveyed with adapted strategies from three therapeutic approaches, which have shown to increase well-being across diagnoses: acceptance and commitment therapy (ACT; [30, 31]) to target mindfulness, acceptance, and valued-based living; dialectical behavioral therapy (DBT; [32]) to enhance emotion regulation, social support, and communication; and compassion-focused therapy (CFT; [33]) to foster a self-compassionate stance. Methods and materials include psychoeducational lectures, experiential exercises and tasks for the transfer to daily life – supported by an accompanying book and a CD demonstrating mindfulness exercises. A detailed description on the conceptualization and implementation of the program has been published in BMC public health [26].

### Participants

The eligible target population were all adult insurance holders of the German insurance fund AOK Baden-Wuerttemberg. The intervention group (IG) was recruited from all members who registered in the Life Balance program between November 2013 and June 2014. Inclusion criteria for the study were: age ≥ 18 years, sufficient German language skills, and capacity to give informed consent. Taking part in the study was optional and was not a precondition for being in the program; thus, the sample was completely self-selected. A total of 1166 participants are included in the cost-effectiveness analyses.

The targeted control group were adult insurance holders not taking part in the intervention. In order to achieve the highest possible level of comparability, study participants in the control group (CG) were recruited in two steps using propensity score matching – a statistical method to build a comparable control group in observational studies [34]. In the first step, a cohort of *n* = 29,482 was selected via propensity score matching (PSM) including potentially relevant covariates that are routinely recorded for all insurance holders (age, sex, health costs, and type of insurance – predominantly employed, family member, retired); and invited to participate in the study only.

In the second step, PSM was used to select a statistical match for all IG and CG participants, for whom cost and psychometric data were available at baseline and the 12-month-follow-up assessment. To achieve optimal comparability of the groups in the primary outcome, we clustered participants by baseline severity of psychopathological symptoms in the HADS [35] using the categorization of no case (≤ 7 points on one of the individual scales), mild [8–10], moderate [11–15], and severe (≥ 16) and imposed a tolerance level of 0.2 on the maximum propensity score distance (caliper; [36]). Matching criteria in the second step were age, sex, self-reported health status and activity, direct and indirect specific as well as unspecific health costs. After matching, the standardized mean difference on all matching variables was < 0.04.

### Data collection

Collection of psychometric data was carried out via mailings prior to the beginning of the program (baseline: t_0_), post-intervention (t_1_ = t_0_ + 10 weeks), and at 6 (t_2_) and 12 (t_3_) month follow-up, in both groups. Health costs were compiled directly from the insurance fund’s records for the duration of the study and – in addition – the 12 months preceding, to map a baseline reference year. Analyses in this article refer to a time-horizon of 12-months, including psychometric data from baseline (t_0_) and the 12-month-follow up (t_3_).

### Health-related outcome

We chose self-reported mental health as primary health outcome, to take into account the specific challenges of detecting change in universal prevention [20], in combination with the ongoing debate about the responsiveness of generic preference-based measures for the assessment of quality-adjusted life-years (QALYs) in mental health (e.g. [37]). The Hospital Anxiety and Depression Scale (HADS, German version) [38] can be considered particularly suitable for use in prevention research, because it displays a high acceptance in non-clinical samples [39], while still yielding a favorable sensitivity and specificity in the clinical diagnosis of depressive disorders (0.82 and 0.74, respectively) [40]. The scale measures symptoms of depression (7-items) and anxiety disorders (7-items) over the past week, using two subscales. Items are rated on a 4-point scale with higher scores indicating higher distress. Its psychometric properties have been validated in numerous samples across age groups, health states and languages [39].

### Cost outcome

All costs are expressed in Euro and were incurred within a 12-month time-horizon, counting from the day the group-sessions started (IG) or the questionnaire was sent in (CG). The reference year (baseline) was mapped by the 12 months preceding that date. Due to the close cooperation with the health insurance fund, it was possible to compile health care costs directly from the insurance fund’s records – and thereby analyze the actual costs that have been spent for each individual study participant within the time-frame of the trial. The health insurance fund’s payment obligations and unit costs for health-care-services (including medication) are highly regulated in Germany and divergences between funds are negligible [41]. Accordingly, the analyzed health care costs were neither weighed nor discounted.

Direct costs comprise all incurred costs for outpatient care, hospital stays, and rehabilitation, which were coded to the diagnoses of mental disorders in the International Classification of Diseases (ICD-10; Chapter V “Mental and behavioural disorders”, F00-F99) and “problems related to life-management difficulty” (ICD-10; Z73) to include stress-related health care utilization, specifically due to the burnout syndrome [42]. Additionally, direct costs include the costs of mental health related medication, i.e. antidepressants, psycholeptics, anxiolytics, sedatives, and hypnotics, as classified by the German Pharmaceutical Atlas [43]. For indirect costs, we included lost work days due to these diagnoses (F00-F99 and Z73), as registered by the cooperating insurance fund. Calculation is based on the human capital approach, by multiplying the lost work days by the loss of gross value added per day of sick leave for the respective years (t_0_ 2014: €105; t_2_ 2015: €109), as estimated by the German Federal Ministry of Labour and Social Affairs [44, 45].

The costs of the intervention were estimated at €93.27 per participant, including developmental and running costs. Developmental costs are composed of conceptualizing, piloting, and training the trainers, and were spread over 5 years, in which 15,000 participants are expected to take part, resulting in net developmental costs per participant of €22.47. Running costs of €70.81 include personnel, rental of practice space, and organization, which will continue to be generated in the future in order to sustain the program.

### Statistical analysis

The economic evaluation was carried out according to the intention-to-treat (ITT) approach. To account for the non-normal distribution of the cost data, means, mean differences, and 95% confidence intervals (95% CI) were obtained by nonparametric bootstrapping with 5000 replications. Differences in health effects, costs, and cost categories between IG and CG were assessed using independent sample t-tests with bootstrapping (5000 replications) – both at baseline and at follow-up, to avoid bias associated with covariates of the propensity score being utilized in subsequent analyses. Incremental cost-effectiveness ratios (ICER) were based on the incremental costs per unit of effect (HADS) and calculated as the difference in the sum of specific direct and indirect costs divided by the inverted difference in HADS score. The inversion was performed to comply with the standard presentation in the cost-effectiveness plane, as improved outcome is associated with lower scores in the original HADS scaling. In the analyses from a health care perspective only direct costs are considered; the societal perspective includes direct and indirect health costs.

Sampling uncertainty in the ICER was handled using nonparametric bootstrapping with 10,000 replications and graphically presented on a cost-effectiveness plane – with incremental effects between IG and CG being depicted on the x-axis and incremental costs on the y-axis. Based on the bootstrapping results, a cost-effectiveness acceptability curve was graphed to determine the probability that the intervention was cost-effective compared to usual care at different willingness-to-pay (WTP) ceiling ratios.

To assess the robustness of the results and account for the large within-group differences in initial distress of participants, we conducted a sensitivity analysis on the severity of psychopathological symptoms in the HADS at the pre-intervention assessment (t_0_) using the categorization of no cases (≤ 7 points on one of the individual scales), mild [8–10], moderate [11–15], and severe (≥ 16) [35]. Analyses were carried out using IBM SPSS 24 with the ICEinfer and PSM package [46, 47]; *p*-values ≤0.05 (two-tailed) were considered statistically significant.

## Results

### Study population

**Fig. 1:**
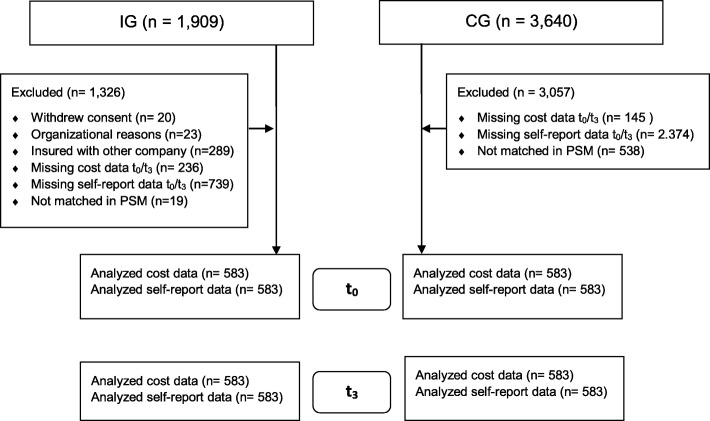
Flowchart of participants

In the IG, 1909 participants agreed to take part in the evaluation, of which 1127 individuals provided complete psychometric data at baseline and 12-months-follow-up. For the economic evaluation, 525 participants had to be excluded because of missing cost data (i.e., were not insured with the cooperating insurance fund consistently across the observation period) and 19 participants were excluded in the PSM (Fig. 1). The included IG participants did not differ from the IG subsample with complete psychometric and cost data at baseline in terms of HADS scores, costs, gender, and marital status, but differed significantly in age (48 vs. 50 years; *p* = 0.001) and education (*p* = 0.005), with the sample described in this article being older and better educated. In the data pool eligible for the CG, 3640 persons agreed to take part in the study. For this analysis, 2374 participants were excluded because of missing psychometric data, 145 because of missing cost data, and 538 in the PSM (Fig. 1). The total study sample of *n* = 1166 participants was mostly female (84%), with a mean age of 50 years (Table 1). At baseline, the sample presented significantly higher scores on the HADS compared to norm values for the general German population (representative population survey; 48).

**Table 1:** Baseline participant’s characteristics

	IG (*n* = 583)	CG (n = 583)	All (*N* = 1166)
Age, years	50.4 ± 12.2	50.5 ± 12.2	50.4 ± 12.2
Female gender	84.9%	83.9%	84.4%
Years of education (%)			
9	33.3%	28.6%	31.0%
10	46.1%	44.4%	45.3%
12+ or 13+	19.9%	26.1%	23.0%
No formal degree	0.7%	0.9%	0.8%
In paid employment	64,5%	66%	65%
Sum score HADS (m ± SD)	15.4 ± 7.0	15.3 ± 7.2	15.3 ± 7.1

### Costs

At baseline, mean total costs were €1458 in the IG and €1436 in the CG, with a statistically insignificant mean difference of €22 (95% CI [− 526, 541], *p* = 0.9; Table 2). At the 12-month follow-up, direct costs were significantly larger in the IG with a mean difference of €181 (95% CI [40, 318], *p* = 0.01; Table 2), which was partly due to the intervention costs of €93 per participant. Indirect costs were lower in the IG (€-237), but this did not reach statistical significance (95% CI [− 749, 236], *p* = 0.34; Table 2).

**Table 2:** Mean annual per-participant costs (in €) and self-reported effects (HADS) by group and assessment time (N = 1166)

	12-Month Baseline				12-Month Follow-Up			
	IG	CG	Differences		IG	CG	Differences	
	Mean (SD)	Mean (SD)	Mean [95%-CI]	p	Mean (SD)	Mean (SD)	Mean [95%-CI]	p
Direct costs	615 (1779)	545 (1614)	69.4 [−122, 272]	0.49	637 (1201)	456 (1318)	181 [40, 318]	0.01
Out-patient care	372 (669)	342 (719)	30.4 [−50, 110]	0.45	431 (797)	337 (708)	94 [10, 180]	0.03
Rehabilitation	45 (432)	12 (200)	33.7 [−2, 77]	0.11	31 (320)	0 (0)	31 [8, 59]	0.07
Medication	32 (100)	35 (125)	−3.1 [−16, 10]	0.63	27 (90)	35 (128)	−9 [−21, 4]	0.18
In-patient care	165 (1,43)	157 (1246)	8.4 [− 147, 168]	0.91	55 (722)	84 (964)	−28 [−131, 64]	0.58
Intervention costs					93 (0)	0 (0)	93 [93, 93]	< .001
Indirect costs	844 (3551)	891 (4074)	−47.4 [− 494, 378]	0.83	749 (3713)	986 (4620)	−237 [−749, 236]	0.34
Total costs	1458 (4523)	1436 (4895)	22.0 [−526, 541]	0.9	1386 (4224)	1443 (5280)	− 57 [− 634, 480]	0.84
Effects (HADS)	15.4 (7.0)	15.3 (7.2)	0.05 [−0.76, 0.87]	0.9	12.4 (6.8)	14.4 (7.7)	−1.97 [−2.81, − 1.14]	< .001

### Cost-effectiveness analyses

**Fig. 2:**
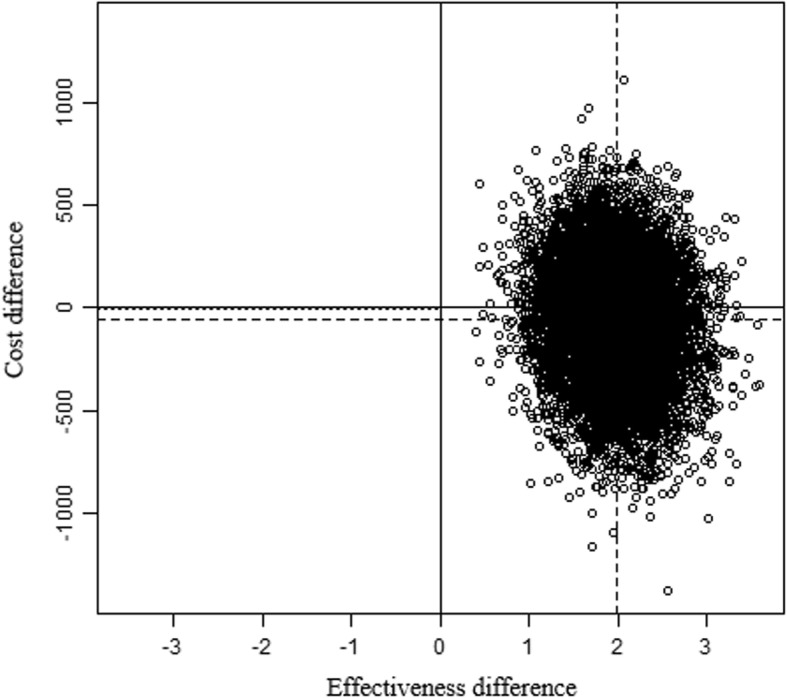
Cost-effectiveness plane; Legend: Units = Cost; Bootstrap Replications = 10.000

**Fig. 3:**
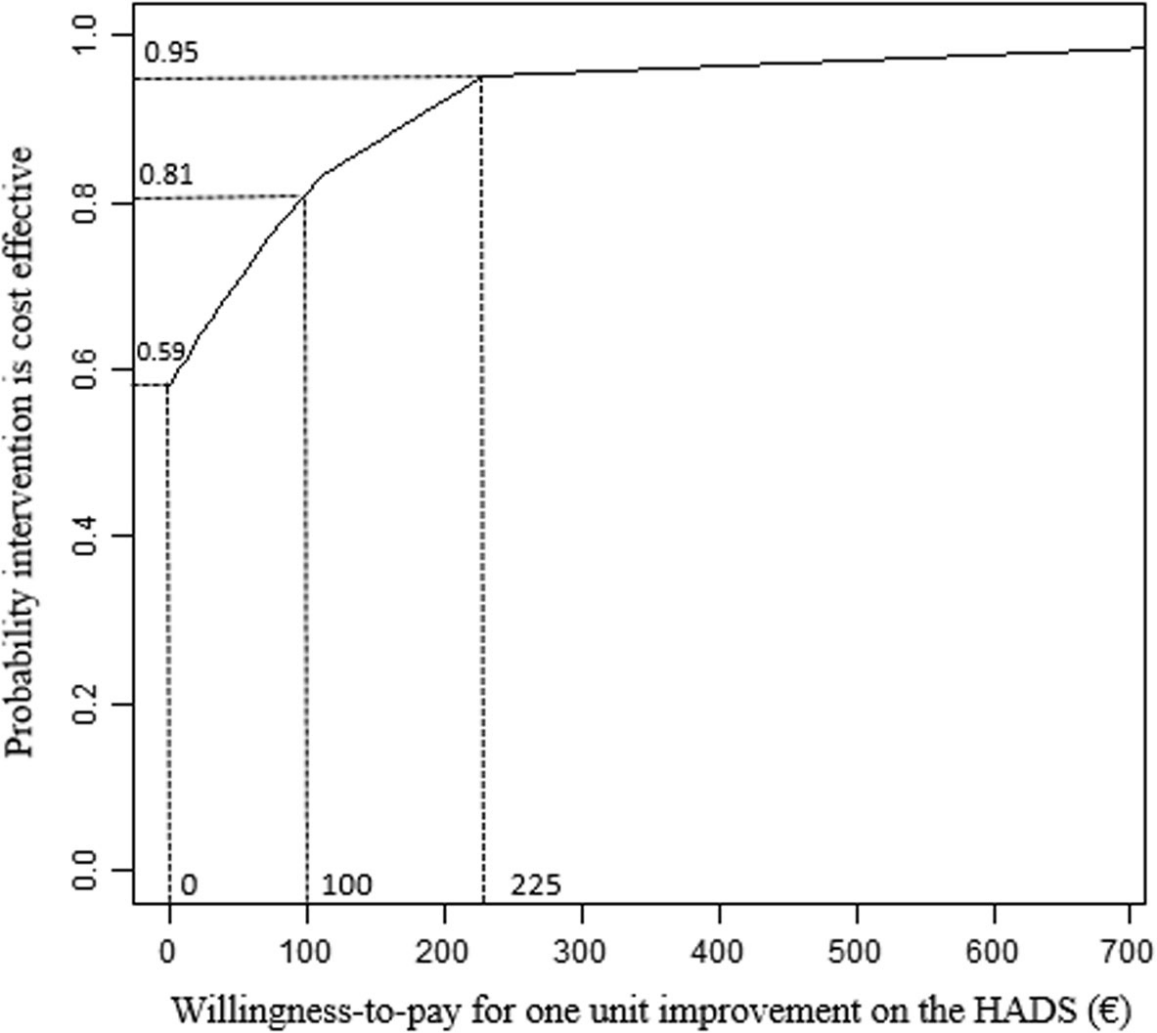
Cost-effectiveness acceptability curve illustrating the probability that the prevention program is cost-effective compared to usual care

From a societal perspective, the intervention had lower bootstrapped mean cost per participant (€-57) and higher mean effects on the HADS (1.97) compared to usual-care, giving rise to an ICER of €-29 (cost savings) per unit improvement on the HADS (see Table 3). On the cost-effectiveness plane, 58.7% of bootstrapped incremental cost/effect pairs were located in the south-east quadrant (see Fig. 2; outcomes improve when moving from left to right) –indicating that the intervention dominated usual care by generating larger health benefits at lower costs. If decision makers were willing to pay €100 per unit of improvement on the HADS, the probabilty for the intervention to be considered cost effective compared to usual care increased to 81%. A maximum willingness-to-pay of €225 was estimated for a 95% probability of cost-effectiveness (see Fig. 3).

**Table 3:** Results of the main and sensitivity analysis

		Cost Difference			Effect Difference^a^				WTP 95% cost effectiveness	%			
Perspective	case	Mean	95%-CI		Mean	95%-CI		ICER		SE^b^	NE^c^	SW^d^	NW^e^
societal	HADS total	−57	−634	480	1.97	1.14	2.81	−29	225	58.7	41.3	0.0	0.0
	no case	300	2	654	1.33	0.36	2.30	225	751	2.2	97.4	0.0	0.3
	mild case	97	− 939	1069	2.13	0.97	3.27	46	474	42.2	57.7	0.0	0.0
	moderate case	− 331	− 1600	861	2.42	1.09	3.72	−137	294	70.6	29.4	0.0	0.0
	severe case	− 1473	− 6274	2665	2.86	−0.59	6.20	−516	1112	70.5	24.7	3.7	1.1
health care	HADS total	181	40	318	1.97	1.14	2.81	91	192	0.9	99.1	0.0	0.0
	no case	98	− 102	273	1.33	0.36	2.30	74	311	14.8	85.0	0.0	0.2
	mild case	253	57	461	2.13	0.97	3.27	119	253	0.7	99.3	0.0	0.0
	moderate case	240	12	469	2.42	1.09	3.72	100	239	2.0	98.0	0.0	0.0
	severe case	37	− 1443	1417	2.86	−0.59	6.20	13	1901	43.8	51.5	2.2	2.5

^a^difference in HADS score inverted (higher score indicating higher improvement)

^b^IG more effective and less expensive than CG

^c^IG more effective and more expensive than CG

^d^IG less effective and less expensive than CG

^e^IG less effective and more expensive than CG

From a health care perspective, the cost-effectiveness plane indicated a probability of 99.1% for the intervention to be more effective, but also more expensive than usual care (north-east quadrant). The ICER was estimated at €91 per unit improvement on the HADS score (Table 3). In other words, an improvement of one unit on the HADS could be realized if decision makers were willing to pay an additional €91 for the intervention. Willingness to pay would need to increase to €192 for a 95% probability of the intervention to be considered cost-effective compared to usual care.

### Sensitivity analyses

In the sensitivity analyses, health effects, costs and sampling uncertainty differed depending on the degree of participants’ initial distress. Across subgroups, the intervention yielded higher bootstrapped mean effects compared to usual-care, ranging from 1.33 to 2.86 units on the HADS (see Table 3). From a health care perspective, these health benefits were consistently achieved at higher costs across subgroups, with largest ICERs for participants initially scoring within the range of mild (€119) and moderate psychological distress (€100). From a societal perspective, the observed effect difference to usual care was associated with higher costs only for initially symptom-free and mildly distressed participants, giving rise to ICERs of €255 (no case) and €46 (mild case), respectively. In contrast, the intervention dominated usual care in participants with higher initial distress with ICERs indicating savings for moderately (€-137) and for severely distressed participants (€-516; Table 3).

For both perspectives taken, cost-effectiveness acceptability curves showed a similar pattern between subgroups in the maximum acceptable cost/effect ratios for a 95% probability of the intervention being cost-effective compared to usual-care. Highest estimates were obtained for the subgroup of severely distressed participants (€1112 societal, €1901 health care), followed by initially symptom-free participant’s (€751 societal, €311 health care) – while willingness-to-pay per unit improvement on the HADS for the same 95% probability was lower in subgroups of mildly (€474 societal, €253 health care) and moderately distressed participants (€294 societal, €239 health care; see Table 3).

## Discussion

The present study analyzed the cost-effectiveness of a mindfulness-based mental health prevention program provided by health coaches in a multi-site field setting on the basis of actually incurred costs. From a societal perspective, the program is likely to be cost-effective compared to usual care, with estimated incremental cost savings of €29 per unit improvement on the HADS for participants of the program (ICER = €-29). From a health care perspective, the intervention was associated with health benefits achieved at higher incremental costs of €91 per unit improvement on the HADS. Cost-effectiveness acceptability curves estimated willingness-to-pay levels of €225 from a societal and €192 from a health care perspective for a 95% probability that the intervention was more cost-effective than usual-care. Sensitivity analyses indicated differences in both, costs and effects, depending on the initial distress of participants.

The multifaceted, complex, and long-term nature of anticipated program benefits of universal prevention for healthy individuals have often been stated as a major reason for the relative shortage of offers and research in universal prevention (e.g. [48]). In line with this debate, results of our sensitivity analyses suggest that one-unit improvement on the HADS requires a higher willingness-to-pay in the subgroup of symptom-free participants than in initially mildly or moderately distressed participants to achieve a 95% probability that the prevention program is cost-effective compared to usual-care. In other words, decision makers have to be willing to pay more for additional health improvements when offering the prevention program to healthy individuals. In the clinical effectiveness evaluation of this program, new cases of psychopathological symptoms were prevented in 1 of 16 participants [28] – which might be an early indicator for potential longer-term benefits in monetary respects. Unfortunately, we did not acquire funding for a longer follow-up period. Large longitudinal studies are needed to appropriately show the effects for initially symptom-free populations.

From a health care perspective, the intervention was associated with incremental costs across subgroups of initial mental distress. Direct costs increased to an average of €181 per participant receiving the intervention. This points at a general issue in universal prevention: Many of the determinants and outcomes of poor mental health lie outside the health sector [48]. The organization that funds a preventive program, in our case a statutory insurance fund, will most likely not profit directly from all its benefits [49]. Consequently, our results underline the call for joint actions and mixed funding paradigms to cope with the challenge of preventing the onset of manifest mental disorders.

The fact that initially moderately and severely distressed participants show the most preferable ICERs from a societal perspective is in line with research on indicated prevention (e.g. [8, 10, 12–14, 16]). Only one of these studies compared indicated and universal prevention. Hunter et al. [11] conclude that identification of risk is likely to be more cost-effective than universal prevention. However, the researchers in this trial did not apply a specifically developed universal prevention program, but instead offered low-intensity depression prevention programs, such as bibliotherapy, online cognitive behavior therapy (CBT) or group therapy. It might, therefore, be possible that the applied programs were not optimally suited for universal prevention. Future research should identify approaches and specific components that are most effective in universal vs. indicative prevention programs [50, 51].

### Strengths and limitations

To our knowledge, this is the first cost-effectiveness analysis of a universal community-based health promotion program. The main strength of this study is the high ecological validity across several domains. The intervention had a large reach and availability for a large public by being offered via face-to-face group sessions on various weekdays and at various times in local health centers located throughout the state. Group sessions were provided by non-specialised health coaches, who continued to work in the program after completion of the evaluation study. Participants were included regardless of their initial psychological distress or other health-related issues, resulting in a quite heterogeneous sample. In future studies, it would be useful to test for unobserved heterogeneity in the intervention sample to identify possible subpopulations that are sensitive to the intervention, as well as who do not respond [52].

A number of possible methodological biases has to be taken into account in the interpretation of our results, many of which relate to the selection and allocation of participants. Due to the complexity of this field trial, it was not feasible to randomize participants and offer an active control condition. This limitation was met by applying propensity score matching (PSM), a rigorous matching procedure which is recommended for the control of the treatment-outcome association in therapeutic studies where randomization is not possible or ethically acceptable [34]. We included all available information as covariates and introduced two self-reported control items to account for potential differences in health-related activities and the willingness to participate in preventive services. Meta-analyses from other medical disciplines indicate that the treatment effects achieved in studies with PSM are comparable or differ only slightly from the effects of randomized-controlled trials: No significant differences were found in trials on surgical procedures [53], a slight underestimation of effect sizes for interventions in critical care medicine [54] and a slight overestimation in the treatment of acute coronary syndromes [55].

As a further measure to reduce the risk of a selection bias, an intent-to-treat-approach was selected for data analyses. Systematic monitoring of attendance rates and attrition from the program were too complex for the means of this study. Informal counting suggests a dropout rate of about 20% of all participants [26], which would be within the range of drop-outs in reviews on health behavior interventions (e.g. [56]). Although no conclusive judgement is possible, it seems reasonable to assume that the ITT analysis at least does not overestimate the effects of the program [57]. Finally, the relatively high nonresponse rate among the population of program participants has to be taken into account in the assessment of a potential selection bias (see [26]). Although it is unlikely that the small differences in age and education of responders compared to non-responders have yielded significant effects on outcome and costs, the results of this study can only be generalized to self-selected participants, who are willing to participate in mental health promotion and the corresponding research without further motivational incentives.

Another potential source of bias in single-study based economic evaluations concerns aspects of cost assessment and valuation [58]. Due to the close cooperation with the health insurance fund, it was possible to obtain actually incurred direct costs and officially registered days of sick leave for the valuing of indirect cost. Any bias stemming from self-reported cost data or estimating health care costs can therefore be excluded [59]. Accordingly, no cost-related bias is expected from the health care perspective. From a societal perspective, this approach can be considered as conservative and might underestimate cost benefits, because a variety of more distal costs are not included in our analyses – such as e.g. costs related to presenteeism [60], productivity losses from unpaid work [12, 13] or informal care [10]. A recent study on indicated prevention of depression, for example, reports the largest indirect savings in terms of presenteeism [12], a societal outcome that was not included in our analysis.

Our choice of the main outcome measure impedes the direct comparison with other economic evaluations reporting costs per quality adjusted life year (QALY) gained [29]. There is an ongoing debate on the suitability, validity and responsiveness of generic preference-based measures for valuing mental health in economic evaluations (e.g. [37]). One possible approach is the development of statistical mapping algorithms using the responses of condition-specific instruments for the estimation of QALYs [61]. However, the development of these algorithm is still in its early stages and requires a strong database to acquire an adequate degree of accuracy. Empirical evidence on the transferability of the HADS shows varying correlations between samples [62–64], and suggests a linear relationship with main deviances at the severe end of the scale [65]. Preliminary analyses based on a linear transformation of the HADS-scores in our sample displayed a willingness-to-pay threshold of around € 9.500 per QALY in the cost-effectiveness acceptability curve to achieve a 95% probability of being regarded as cost-effective. Although this number seems to be within a reasonable range compared to cost-effectiveness-analyses of indicated prevention (e.g. [12]), it can at most serve as a first impression of possible effects to stimulate future research on universal primary prevention.

### Implications

Policy makers and insurance companies have to fill the gap between the pleas for the promotion of mental health and the prevention of mental disorders (e.g. [6, 66]) and limited health care budgets. Informed decision making requires data on the cost-effectiveness of possible interventions. Economic evaluations of real-world prevention strategies are rare, largely because of the associated methodological challenges and high research costs (e.g. [48]). The data presented in this article give a first impression that universal mental health prevention programs for adults in a population setting might be a cost-effective strategy to enhance well-being. However, further research is needed to enhance large-scale implementation of such programs. While this study evaluated a mindfulness-based intervention, most studies regarding the cost-effectiveness of selective and indicated interventions are based on CBT (e.g. [7]). Large trials are necessary to compare different approaches and isolate the active components of interventions that might facilitate stronger outcomes and superior cost benefits [50].

Longitudinal research on more distant parameters might further emphasize the societal relevance of easily accessible universal prevention. Better mental health has been associated with improved outcomes in a range of other domains, such as physical health, health behaviors, education, and earnings or crime reductions (e.g. [67]), which are mostly not assessed in cost-effectiveness analyses due to the time lag of effects. The societal benefit of health promotion might thus be underestimated in shorter-term studies, such as ours.

Furthermore, this study adds to the growing evidence that trained laypersons with no prior professional mental health training can effectively be involved in health promotion and treatment of subsyndromal and mild mental disorders. The role of these non-specialist health workers is that of a coach, following structured intervention protocols, as opposed to a traditional therapist role [68]. Non-specialist health workers are of particular importance in low- and middle-income countries where human resources for mental health are scarce (e.g. [69]), but can also contribute to the affordability of mental health promotion in high-income countries by reducing costs for highly trained professionals. Recent research on the British “Improving the Access to Psychological Therapies” program shows a complex, non-linear relationship between non-specialist health workers’ competence and patient outcome [70]. More research is needed to investigate the service of health workers in preventive interventions in industrialized countries.

## Conclusions

In conclusion, our data show that the Life Balance intervention was not only effective in reducing mental distress and future risk of psychiatric disorders but also yielded favorable cost-effectiveness ratios from a societal perspective. Differences between the societal and health care perspective underline the call for joint funding in the dissemination of preventive services.

## Data Availability

The datasets generated and/or analysed during the current study are not publicly available due to the data protection policy from the cooperating insurance fund but are available from the corresponding author on reasonable request (in anonymized form).

## Abbreviations

ACT: Acceptance and commitment therapy
CBT: Cognitive-behavioral therapy
CFT: Compassion-focused therapy
CG: Control group
CI: Confidence interval
DBT: Dialectical behavioral therapy
HADS: Hospital Anxiety and Depression Scale
ICD: International Classification of Diseases
ICER: Incremental cost-effectiveness ratio
IG: Intervention group
ITT: Intention-to-treat
PSM: Propensity score matching
QALY: Quality-adjusted life year
WTP: Willingness-to-pay
